# Investigation of Size Effects in Multi-Stage Cold Forming of Metallic Micro Parts from Sheet Metal

**DOI:** 10.3390/mi12121561

**Published:** 2021-12-15

**Authors:** Martin Kraus, Marion Merklein

**Affiliations:** Department of Mechanical Engineering, Institute of Manufacturing Technology, Friedrich-Alexander-Universität Erlangen-Nürnberg (FAU), Egerlandstr. 13, 91058 Erlangen, Germany; Marion.Merklein@fau.de

**Keywords:** size effects, microforming, accumulative roll bonding (ARB), ultrafine grain structure

## Abstract

Product miniaturisation and functional integration are currently global trends to save weight, space, materials and costs. This leads to an increasing demand for metallic micro components. Thus, the development of appropriate production technologies is in the focus of current research activities. Due to its efficiency, accuracy and short cycle times, microforming at room temperature offers the potential to meet the steadily increasing demand. During microforming, size effects occur which negatively affect the part quality, process stability, tool life and handling. Within this contribution, a multi-stage bulk microforming process from sheet metal is investigated for the materials Cu-OFE and AA6014 with regard to the basic feasibility and the occurrence of size effects. The results reveal that the process chain is basically suitable to produce metallic micro parts with a high repeatability. Size effects are identified during the process. Since several studies postulate that size effects can be minimised by scaling down the metallic grain structure, the grain size of the aluminium material AA6014-W is scaled down to less than one micrometre by using an accumulative roll bonding process (ARB). Subsequently, the effects of the ultrafine grain (UFG) structure on the forming process are analysed. It could be shown that a strengthened material state increases the material utilization. Furthermore, too soft materials can cause damage on the part during ejection. The occurring size effects cannot be eliminated by reducing the grain size.

## 1. Introduction

The demand for metallic micro parts is growing due to a steady miniaturisation with a simultaneous functional integration [1]. These parts are widely applied in the fields of electronics, automotive, micro electromechanical systems, microsystems technology and medical technology [2]. Currently, metallic micro parts are primarily fabricated by the lithographic fabrication technology (LIGA) [3], micropowder injection moulding [4], micromachining [5] and microforming [6]. To meet the great demand, manufacturing processes, which are capable to produce the micro parts in high quantities at short cycle times in a high quality, repeatable and cost-efficient, are required. In mass production of metallic parts, forming technology offers economical, technological and ecological advantages in comparison to other production technologies. Nevertheless, the scaling of forming processes into micro dimensions leads to size effects [7]. 

These are especially challenging for bulk microforming processes due to the complex handling. When scaling down the part size, the ratio of the surface area to the part volume increases quadratically [8]. This results above a certain part size in stronger adhesion forces, magnetism and Van der Waals forces than the gravity force and thus, the parts stick to the handling devices. In addition, the small part size complicates the exact positioning in the dies during the production process. For this reason, scientists are working on solutions to improve the handling of the micro parts during multi-stage forming. Arentoft et al. have developed an inline part-transfer system in a multi-stage bulk micro forming process, which enables a production rate of 50 parts per minute [9]. Hirota [10] has investigated the direct extrusion of metallic micro parts from sheet metal in order to simplify the handling considerably. In this process, the sheet metal serves both as a semi-finished product and as a component carrier between the multiple forming stages. This process strategy has already been investigated in numerous research studies on different materials. For example, Ghassemali et al. analysed the influence of different punch/die ratios on the achievable height/diameter ratios of the pins [11]. Meng et al. [12] investigated this new process class for the fabrication of flanged micro parts from copper sheet in a continuous process. In their work, the influences of different grain sizes on the forming and cutting edge have been analysed. The handling of sheet metal strip has already been established in industrial sheet metal microforming processes. For this reason, scaling this strategy into mass production appears feasible.

In this paper, this strategy is further investigated by analysing the whole process chain of a multi-stage bulk microforming process from the sheet metal to the finished micro part. The focus of the study is to prove the basic feasibility of the process chain. Furthermore, size effects are identified by studying the process in both macro and micro scale. The transferability of the findings is guaranteed by the use of the two different materials Cu-OFE and AA6014. Numerous scientific studies prove that size effects can be minimised by using ultrafine grain structures. Rosochowski et al. already showed in 2007, by using micro-cup extrusion tests, that the use of nano-crystalline microstructures can improve the homogeneity of the material flow and the surface quality in bulk microforming processes [13]. Dahl et al. have proven in deep drawing tests that compared to conventional grain structures, the use of nano-crystalline grain structures can significantly improve the surface quality, the dimensional stability and the process scattering in micro sheet forming [14]. Thus, the microstructure of the material AA6014 is modified to this state by using an accumulative roll bonding (ARB) process. The ARB process is capable to produce ultra-fine grain structures by rolling the material in several layers and to increase the material strength considerably [15]. Subsequently, micro scale tests are carried out again with this material state. Afterwards, the impact on the size effects and the potential of UFG materials during this process are evaluated.

## 2. Experimental Setup

**Figure 1:**
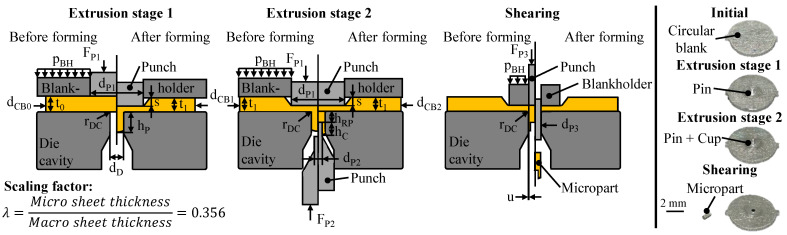
Investigated process chain with resulting part geometries of the different forming stages.

The used three-stage process chain with the resulting geometry of each forming stage is shown in Figure 1. All relevant process parameters are given in Table 1. For the tests, a multi-acting tool system was used which allows a separate control of the blankholder and the upper and lower punch. The different process stages can be implemented on the tool system by using interchangeable active elements. The tool was installed in a universal testing machine Z100 (Zwick/Roell GmbH, Ulm, Germany). The testing machine controls the blankholder. The punches are driven by two hydraulic cylinders (AHP Merkle GmbH, Gottenheim, Germany) installed in the tooling system. For the identification of size effects, the process is investigated in the macro and micro scale for Cu-OFE by using the scaling factor of *λ* = 0.356. The blankholder pressure is set material-specific close to the yield stress of the material being tested. This ensures the highest possible material flow barrier without a significant plastic compressing of the sample [16]. For lubrication, the extrusion oil Dianol ST is used in a ratio of 10 g/m^2^. The forming speed was set to 5 mm/min for all forming stages. In order to exclude any heat influence or geometric inaccuracies during test specimen fabrication, the circular blanks were manufactured by using micro waterjet cutting.

In extrusion stage 1, material is accumulated in the form of a pin. This pin provides the material for the subsequent forming stages. In this forming stage, the tooling system consists of a die, a punch and a blankholder. At the process start, the workpiece is axially constrained by the blankholder to prevent the sheet metal from bulging during extrusion. Afterwards, the punch moves downwards, resulting in a desired material flow axially and radially towards the die cavity as well as an unwanted material flow radially outwards into the sheet metal plane.

During extrusion stage 2, a cup with different diameters is formed on the pin from extrusion stage 1. For this purpose, the tool system remains closed after pin forming. During cup extrusion, a second punch is moved axially upwards until the desired restpin height is reached. This results in a targeted material flow towards the cup wall as well as an undesired material flow back into the sheet metal plane.

In the third process stage, the micro part is separated from the sheet metal by shearing. Here, the tool consists of an adapted blankholder and a cutting punch. After axial fixing of the sheet, the cutting punch moves downwards. As a result, the micro part is cut off from the carrier sheet and ejected downwards. 

**Figure 2:**
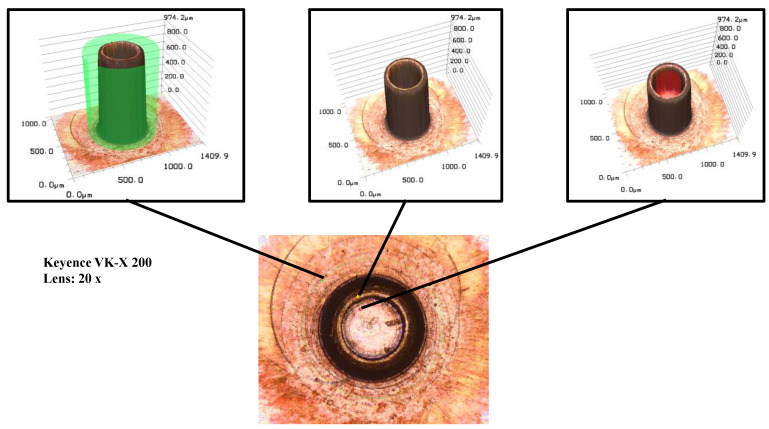
Strategy for measuring the micro parts by using a confocal laser scanning microscope.

Due to the small dimensions of the micro parts, conventional tactile measuring devices such as micrometres or callipers cannot be used for measurement. In order to ensure the required measurement accuracy, all components were scanned using the Keyence VK-X 200 confocal laser scanning microscope (KEYENCE Deutschland GmbH, Neu-Isenburg, Germany) in a temperature-controlled laboratory at 20 °C. A lens with 20× magnification was used for the measurement. This lens is also suitable for an optical determination of the surface characteristics. To determine the pin and cup heights, the heights of the individual layers were determined by using 3-point circles, as shown in Figure 2. By adding or subtracting the height values, the required component sizes can be determined.

For the determination of the roughness values, the curved component topography was first extracted by means of a coordinate transformation using the VK-Analyze-Module software (KEYENCE Deutschland GmbH, Neu-Isenburg, Germany). Subsequently, ten parallel lines were drawn along the micro part on the flat surface to record the roughness of the lines. The roughness parameters were then determined by using the roughness profile of the individual lines in accordance with the DIN EN ISO 4287 standard.

## 3. Materials

The materials Cu-OFE and AA6014 are used in the experimental tests. The composition of the two materials is given in Table 2. Due to its high purity and the single-phase microstructure, the copper material is excellently suited to investigate size effects. Furthermore, it has a high application potential for electronic micro components, such as connector pins, due to its high conductivity. To achieve a homogeneous grain structure, the material is heat treated at 650 °C for one hour. The heat treatment results in an identical grain size for the copper material in macro and micro scale. The used precipitation hardenable aluminium alloy AA6014 is interesting for applications in microforming technology due to its corrosion resistance and low density combined with a high strength. A potential application would be, for example, drive shafts for micro motors or micro gears. For studying size effects, the aluminium alloy is investigated in two different states. AA6014-W is solution heat treated at 545 °C for 15 min. For AA6014-ARB, accumulative roll bonding is used to achieve an ultrafine grain structure. For this purpose, the ARB-process chain, described in Hermann et al. [17], is used with four repetitions.

**Figure 3:**
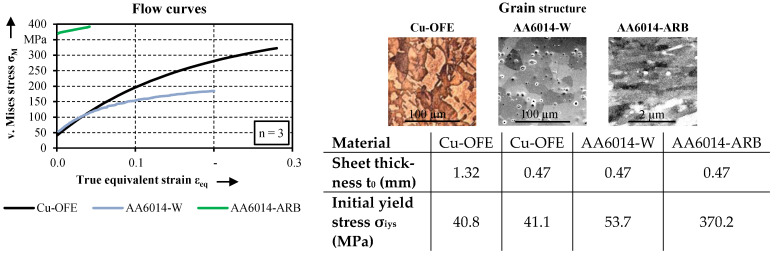
Flow curves, grain structure and initial yield stress of the used materials.

Figure 3 shows the mechanical properties of the used materials based on the flow curve and the initial yield stress. The flow curves were obtained by uniaxial tensile tests according to DIN EN ISO 6892-1 in rolling direction. Furthermore, the grain structures are shown by micrographs. For the determination of the grain size, the samples were etched and micrographs were recorded by using light microscopy (Cu-OFE) and the scanning electron microscope Merlin with Gemini 2 electron optics (Zeiss; Jena). The grain size was determined according to the standard DIN EN ISO 643:2013-05. The average grain size in rolling direction is 41 µm for Cu-OFE [18], 21 µm for AA6014-W and 0.64 µm for AA6014-ARB [17].

## 4. Results

### 4.1. Extrusion Stage 1 (Pin-Forming)

**Figure 4:**
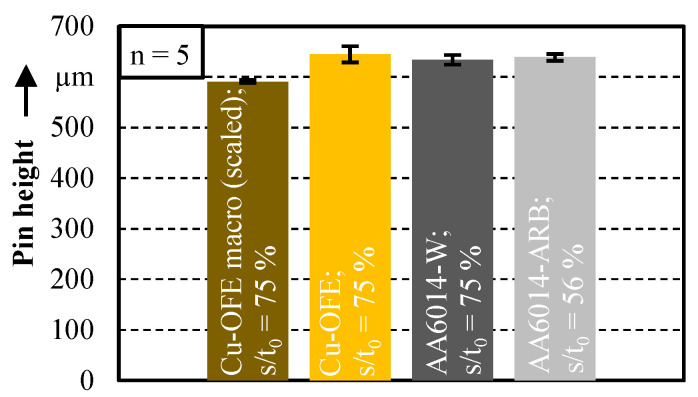
Pin heights after extrusion stage 1 for macro (scaled) and micro scale.

In Figure 4, the achieved pin heights after extrusion stage 1 are presented for the macro and micro scale. For an improved comparability of the results, the original pin height *h* = 1.660 mm of the macro experiments is scaled in the diagram with the scaling factor *λ* = 0.356. 

It is noticeable that the pin heights between macro and micro scale are at a comparable level. The slightly lower pin height for the macro scale is attributed to manufacturing inaccuracies in the area of the die entry radius and not to size effects. The material utilisation for Cu-OFE is 18.3% in the macro and 19.8% in the micro scale. The standard deviation is slightly larger in the micro scale. However, with a maximum standard deviation of <2.5%, the pin extrusion process can be classified as repeatable, even in the micro scale. In micro scale, a relative penetration depth of *s*/*t*_0_ = 75% for the material AA6014-W (*h* = 634 µm) results in a comparable pin height as for Cu-OFE (*h* = 645 µm). The pin height for the material AA6014-ARB is significantly larger for the identical penetration depth. Since this pin height would exceed the cylindrical length of 1 mm of the die used in extrusion stage 2, an identical pin height to the AA6014-W material is set for the AA6014-ARB material. Here, the pin height of 639 µm is already achieved at a relative punch penetration depth of *s*/*t*_0_ = 56%. Thus, the material utilisation of AA6014-ARB is 26.8% at this penetration depth. According to the findings of Ghassemali et al. [11], the utilisation rises further with an increasing punch penetration depth. The higher material utilisation can be traced back to the significantly increased strength of the ARB material. This effect has been already experimentally observed by Hirota and Michitsuji [19] for the aluminium A1050 and by Merklein et al. [20] for copper materials.

### 4.2. Extrusion Stage 2 (Cup-Forming)

**Figure 5:**
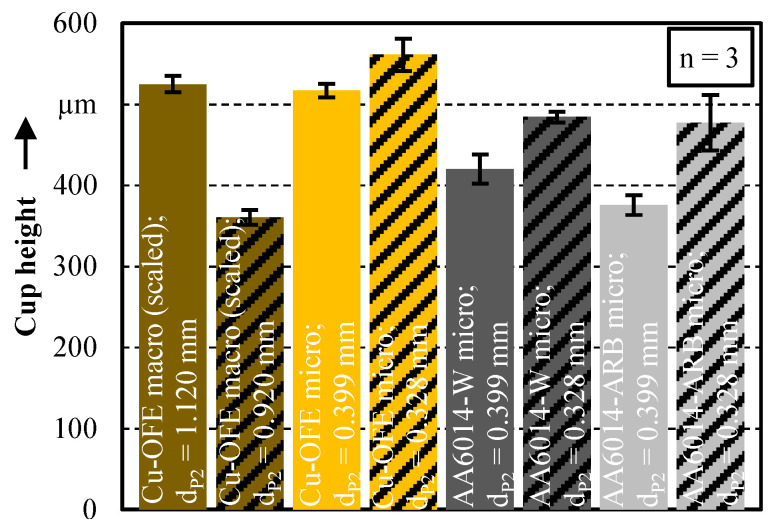
Resulting cup heights in macro (scaled) and micro scale after extrusion stage 2.

The achieved cup heights for the different materials in relation to the wall thickness are compared in Figure 5. Also here, the original cup heights *h*_1.120_ = 1.475 mm and *h*_0.920_ = 1.013 mm in the macro scale are scaled down with the factor of *λ* = 0.356 for an improved comparability. It is noticeable that the cup height in the macro scale is higher for the smaller wall thickness (wt). This can be explained by the fact that a larger punch diameter is used for the smaller wall thickness of 100 µm, which displaces more material. For the smaller wall thickness, the circular ring area of the cup is also significantly smaller. This results in higher cup heights for the same material volume. The opposite applies to the micro scale. Due to a size effect, the cup height is higher for the thicker wall thickness of 71 µm. This size effect is also present for the materials AA6014-W and AA6014-ARB.

**Figure 6:**
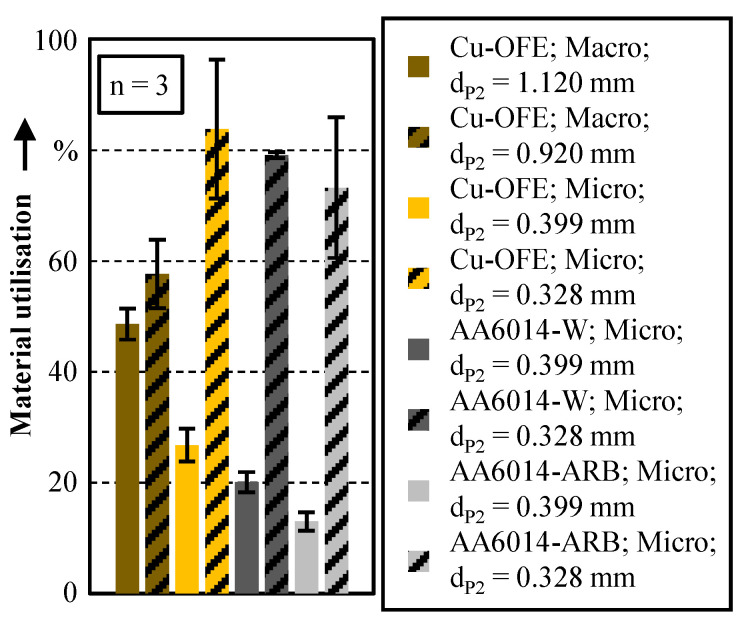
Material utilisation during extrusion stage 2.

For a more detailed analysis of this size effect, the material utilisation of extrusion stage 2, shown in Figure 6 by the ratio of the displaced material volume in the cup to the displaced material volume by the die, is considered. First, it can be observed that the material utilisation is higher for the micro as well as for the macro scale, for the bigger wall thicknesses. This can be attributed to the enlarged flow gap and the associated lower deformation resistance due to the higher distance between the two friction surfaces, punch and die. In the macro scale, the material utilisation of 48.6% for wt = 100 µm and 57.6% for wt = 200 µm is only slightly different. This can be explained by the identical flow resistance in the sheet metal plane between punch/die and blankholder/die for both wall thicknesses. For the thinner wall thickness of 100 µm, the flow resistance is a little bit higher. However, the flow gap of 100 µm is large enough to displace the material into the cup wall without a significant increase in the deformation resistance.

In contrast, there is, with an average difference of 60% between wt = 71 µm and wt = 36 µm, a high dependency of the material utilisation on the wall thickness in the micro scale. By using the smaller punch diameter of *d*_P2_ = 0.328 mm, the material utilisation increases unexpectedly in comparison to the macroscopic process. This anomaly can be explained by the grain structure after extrusion stage 1. In the pin head, the initial grain structure is nearly undeformed in both the macro and micro scale [18]. In the micro scale, the number of grains over the cross section is significantly lower. Thus, fewer dislocations can be accumulated at the grain boundaries. In accordance to the Hall–Patch relation, the hardening and also the strength is therefore lower in the micro than in the macro scale. This assumption can be also confirmed by the microhardness measurements of the pin in Kraus et al. [12]. Here, the pin head shows a lower hardness in the micro scale. This is why the material can flow more easily into the cup wall. Thus, both the material utilisation and the pin height are significantly higher for *d*_P2_ = 0.328 mm compared to the larger wall thickness in macro scale.

A further size effect is the significantly lower material utilisation (Figure 6) and cup height (Figure 5) for the smaller cup wall thickness of 36 µm (*d*_P2_ = 0.399 mm) in the micro scale. Actually, the lower material utilisation can be well explained by the in the state-of-the-art known surface layer model. Wang et al. [2] has applied this model for bonded forming in a comparable micro-coining process. Here, it is postulated that the friction inhibited the material flow via the surface grains. With a decreasing number of grains, an increase of the flow resistance is assumed. Once a grain count of two is reached, all grains are constrained in movement by the tools and the flow resistance reaches its maximum. 

**Figure 7:**
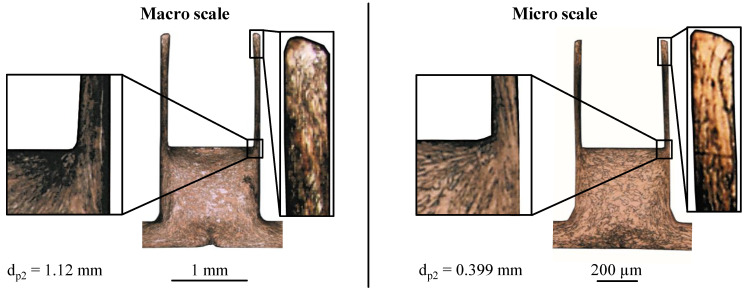
Micrographs of the cup cross-section after extrusion stage 2 for Cu-OFE in the macro and micro scale.

For the materials Cu-OFE and AA6014-W, this model fits to explain the low material utilisation at *d*_P2_ = 0.399 µm, since there are just 1–5 grains across the cup cross-section (Figure 7). The micrographs show that in macro scale the grains are highly stretched in the direction towards the cup wall (flow lines). This indicates an increased material flow into the cup wall. In the micro scale, the grains are considerably less deformed due to the high deformation resistance caused by the low grain count, which indicates a reduced material flow into the cup wall. In order to verify the surface layer model in this process, additional tests are carried out with the ultrafine grain material AA6014-ARB. Due to its grain size, at least 58 grains are present over the smallest wall thickness of wt = 36 µm. Thus, according to the surface layer model, more material should flow into the cup wall due to the relatively small proportion of surface grains. Nevertheless, the size effect of a significant decrease in material utilisation in extrusion stage 2 can be observed here as well. Considering the results with the different materials and grain sizes, it can be interpreted that the increasing flow resistance in the cup is not significantly dependent on the grain size, but mainly on the width of the flow gap and possibly on the material strength. This independence of the material utilisation from the grain size in a geometrical correctly scaled process leads to the conclusion that the laws for calculating the deformation resistance are not valid for very small wall thicknesses. A possible explanation for this might be the very small flow gap between the two tool surfaces. Due to the large ratio between tool contact surface and workpiece volume, the material flow is heavily inhibited by the wall friction. This effect has already been detected by Deng et al. [21] in scaled upsetting tests and justified in a similar way. The higher the material strength, the more wall friction can be transferred. Thus, the material utilisation decreases with an increasing material strength. This assumption is confirmed for the materials Cu-OFE and AA6014-W in Figure 6. Despite the decreasing material utilisation, the material AA6014-ARB cannot be used to verify this theory since the die penetration depth in extrusion stage 1 is 19% lower. Thus, the flow resistance between punch/blankholder and die is lower, which might be also a factor for the lower material utilisation of the ultrafine grained material.

The standard deviation for the cup forming in extrusion stage 2 (Figure 5) is slightly larger than for the pin forming process. With a maximum standard deviation of <2.5% in the macro scale (Cu-OFE; *d*_p2_ = 0.920) and 7.2% in the micro scale (AA6014-ARB; *d*_p2_ = 0.328), the pin extrusion process can still be classified as repeatable, even in the micro scale. The common rolled copper and aluminum materials show a lower maximum standard deviation of 4.3% (AA6014-W; *d*_p2_ = 0.399) in the micro scale.

### 4.3. Shearing (Separation of the Micro Part)

**Figure 8:**
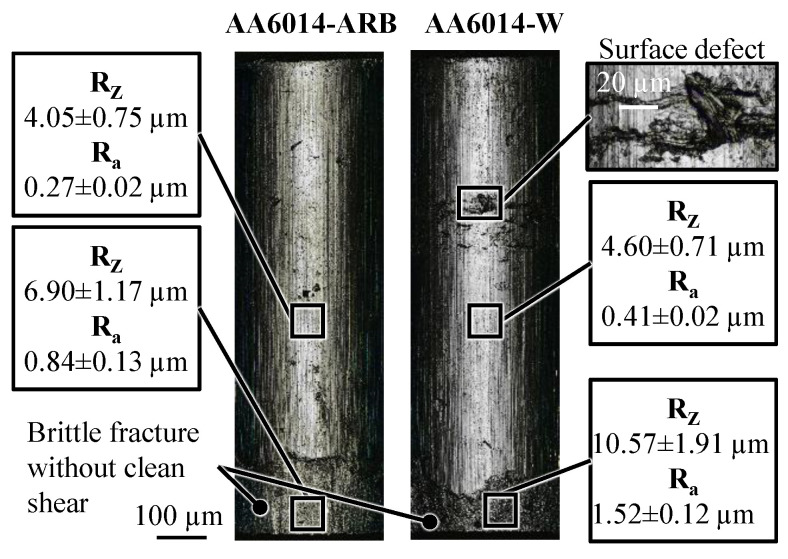
Comparison of the surface and component quality of the micro parts made of AA6014-W and AA6014-ARB.

To evaluate the cutting edge after shear cutting and the surface quality of the parts, the surface topography is analysed by confocal laser scanning microscopy on a Keyence VK-X 200. A 50× magnification lens is used to achieve a high resolution. The surface images for the materials AA6014-W and AA6014-ARB are presented in Figure 8. Comparing the two components, a damage of the surface of the micro part made of AA6014-W is noticeable. This damage is located in the area of the transition between the cup wall and the remaining pin. This can be related to the ejection process after extrusion stage 2. During ejection, the micro part is subjected to tensile stress due to the wall friction. As a result of the reduction of the cross-section in the pin/cup wall transition zone, particularly high tensile stresses occur in this area. Thus, the considerably lower material strength of AA6014-W leads to damage to the cup wall. For this reason, materials with the highest possible strength are recommended for a stable process.

For both materials, the cutting edges show a brittle fracture without a clean-shear area. This is caused by the high work hardening in the pin foot area during pin forming in extrusion stage 1. As a result of the lower punch penetration depth, the fracture zone for AA6014-ARB is longer than for AA6014-W. For AA6014-ARB, the cutting edge shows a slightly smoother fracture surface overall. This can be explained by a more brittle fracture due to the higher pre-hardening. The component surface is at a comparably good level for both materials with R_Z_ 4.05 µm for AA6014-ARB and R_Z_ 4.60 µm for AA6014-W. With this roughness, the micro parts are in the range of the achievable surface quality for formed aluminium parts.

## 5. Summary and Outlook

In this article, multi-stage bulk forming of cylindrical micro parts with cup from sheet metal is investigated. It has been proven that the 3-stage forming from the sheet metal to the finished micro part can be performed with a high reproducibility. The process scaling into the micro scale leads to an occurrence of size effects. Depending on the geometry, these effects improve or reduce the material utilisation. It has been shown that these size effects cannot be reduced or even prevented by using ultrafine grain structure materials. Furthermore, it is evident that the grain size has no measurable influence on the material flow. In the micro scale, the material utilisation decreases for low wall thicknesses due to the increasing ratio between tool contact area and component volume. Besides the changed geometrical aspect ratio, only the strength of the material seems to influence the die filling. During ejection of the pin with cup after backward extrusion, damage to the cup surface occurs in soft material conditions due to high tensile stresses in the cup area. For this reason, high-strength materials are recommended for the forming of filigree, large-area form elements in the micro scale.

Future research work will focus on the transferability of the successful laboratory tests to mass production. In this context, it should be demonstrated that the micro parts can be fabricated in a progressive die on a high-speed press in high quantities with a high repeat accuracy.

## Figures and Tables

**Table 1:** Process parameters for cold bulk macro- and microforming from sheet metal.

Parameters		Unit	Macro	Micro
Relative punch stroke	*s*/*t*_0_	(%)	75 (Cu-OFE; AA6014-W); 56 (AA6014-ARB)	
Blankholder pressure	*p*_BH_	(MPa)	50 MPa (Cu-OFE); 70 MPa (AA6014-W); 300 MPa (AA6014-ARB)	
Sheet thickness	*t*_0_	(mm)	1.32	0.47
Blank diameter	*d*_CB0_	(mm)	15.00	5.34
Restpin height	*h*_RP_	(mm)	1.000	0.356
Punch diameter 1	*d*_P1_	(mm)	4.00	1.42
Punch diameter 2	*d*_P2_	(mm)	0.920; 1.120	0.328; 0.399
Punch diameter 3	*d*_P3_	(mm)	-	0.43
Die diameter	*d*_D_	(mm)	1.32	0.47
Die radius	*r*_DC_	(mm)	0.14	0.05

**Table 2:** Chemical composition of the used materials.

	**Cu-OFE**							
**Alloying Element**	**Cu**							
Chemical composition (%)	min. 99.99							
	**AA6014**							
**Alloying Element**	**Si**	**Fe**	**Cu**	**Mn**	**Mg**	**Cr**	**Zn**	**Ti**
Chemical composition (%)	0.30–0.60	max. 0.35	max. 0.25	0.05–0.20	0.40–0.80	max. 0.20	max. 0.10	max. 0.10
